# Case Report: Solitary fibrous tumor (SFT) of cerebellum: report of 2 cases

**DOI:** 10.3389/fsurg.2026.1944873

**Published:** 2026-10-14

**Authors:** Gaochao Guo, Zongzong Ma, Liming Zhao, Tao Gao, Yuxue Sun, Ziqiang Liu, Yang Liu, Chaoyue Li

**Affiliations:** 1 Department of Neurosurgery, Henan Provincial People’s Hospital, Zhengzhou, China; 2 Department of Neurosurgery, Nanyang Nanshi Hospital, Nanyang, China

**Keywords:** cerebellum, prognosis, radiotherapy, solitary fibrous tumors (SFTs), surgical excision

## Abstract

**Objective:**

To elucidate the neuroradiological characteristics, clinical manifestations, and surgical outcomes of intracranial cerebellar solitary fibrous tumors (SFTs) to improve diagnostic accuracy and therapeutic strategies.

**Method:**

A retrospective analysis was conducted on the clinical data of two histopathologically confirmed cases of cerebellar SFT, supplemented by a review of the relevant literature.

**Results:**

Both patients underwent total surgical excision followed by postoperative radiotherapy. At the latest follow-up, neither patient showed radiological evidence of tumor recurrence (Case 1: 18 months; Case 2: 12 months]). Given the potential for late recurrence in SFTs, long-term surveillance is mandatory.

**Conclusion:**

Intracranial cerebellar SFTs are rare neoplasms that are frequently misdiagnosed despite exhibiting distinctive magnetic resonance imaging (MRI) features. Definitive diagnosis relies on histopathological and immunohistochemical analyses. In this study, MRI demonstrated T1-weighted imaging (T1WI) hypointensity or isointensity, T2-weighted imaging (T2WI) heterogeneity, and inconsistent post-contrast enhancement. Immunohistochemical evaluation revealed positivity for CD34, CD99, and STAT6. Prompt surgical intervention accompanied by adjuvant therapy was associated with a favorable prognosis.

## Introduction

Intracranial solitary fibrous tumor (SFT) is a rare spindle cell neoplasm of mesenchymal origin that most commonly occurs in serosal surfaces. Intracranial occurrence is exceedingly rare, accounting for approximately 1% of all primary central nervous system tumors (1). Owing to variations in lesion sites and pathological grades, clinical and imaging manifestations differ significantly. Consequently, the clinical presentation often mimics that of other intracranial tumors, frequently leading to preoperative misdiagnosis. Although SFTs have been increasingly reported in recent years, their occurrence in the cerebellum is highly unusual and frequently leads to preoperative misdiagnosis. The two cases presented here are instructive in three respects: they illustrate the characteristic imaging features that should raise suspicion for SFT; they demonstrate the central role of nuclear STAT6 immunostaining in confirming the diagnosis; and they exemplify a risk-adapted postoperative management strategy for high-grade lesions.

### Clinical data

Patient 1 was a 45-year-old male admitted to our hospital on March 04, 2023, with a one-month history of dizziness and a cerebellar space-occupying lesion identified 3 days before admission. Physical examination demonstrated intact consciousness, isocoric and round pupils with brisk light reflexes, normal muscle strength and tone in all four limbs, absence of pathological signs, and negative meningeal irritation signs. Magnetic resonance imaging (MRI) revealed a well-circumscribed, spherical lesion in the right cerebellar hemisphere, showing a slightly hypointense signal on T1-weighted imaging (T1WI) and a heterogeneous signal on T2-weighted imaging (T2WI) (Figures 1A,B). Post-contrast gadolinium-diethylenetriaminepentaacetic acid (Gd-DTPA) enhancement demonstrated significant lesion enhancement with several irregular focal areas of enhancement in the center, locally connected to the meninges (Figure 1C). The preliminary diagnosis was a space-occupying lesion in the right cerebellar hemisphere, with meningioma considered as a differential diagnosis. Intraoperatively, the lesion base in the subtentorial region was found to be closely adhered to the cerebellar tentorium and transverse sinus. The lesion was characterized by clear boundaries, complete encapsulation, a firm texture, and a rich vascular supply. Histopathological examination confirmed a SFT, classified as WHO Grade 3 based on the presence of tumor necrosis and mitotic activity exceeding 5 mitoses per 10 high-power fields. Immunohistochemical staining showed positivity for CD34, STAT6, Syn, and CD99, with a Ki-67 (MIB-1) proliferation index of approximately 5%. The tumor was negative for NSE, EMA, GFAP, and PR (Figures 1D,E; Supplementary Document 1). The Demicco risk stratification system score was 3, classifying the patient in the low-risk category (Table 1; Supplementary Documents 1, 2). The patient underwent adjuvant radiotherapy one month postoperatively. Clinical and radiological follow-up over a 1.5-year period demonstrated no evidence of tumor recurrence (Figures 1F–H).

**Figure 1:**
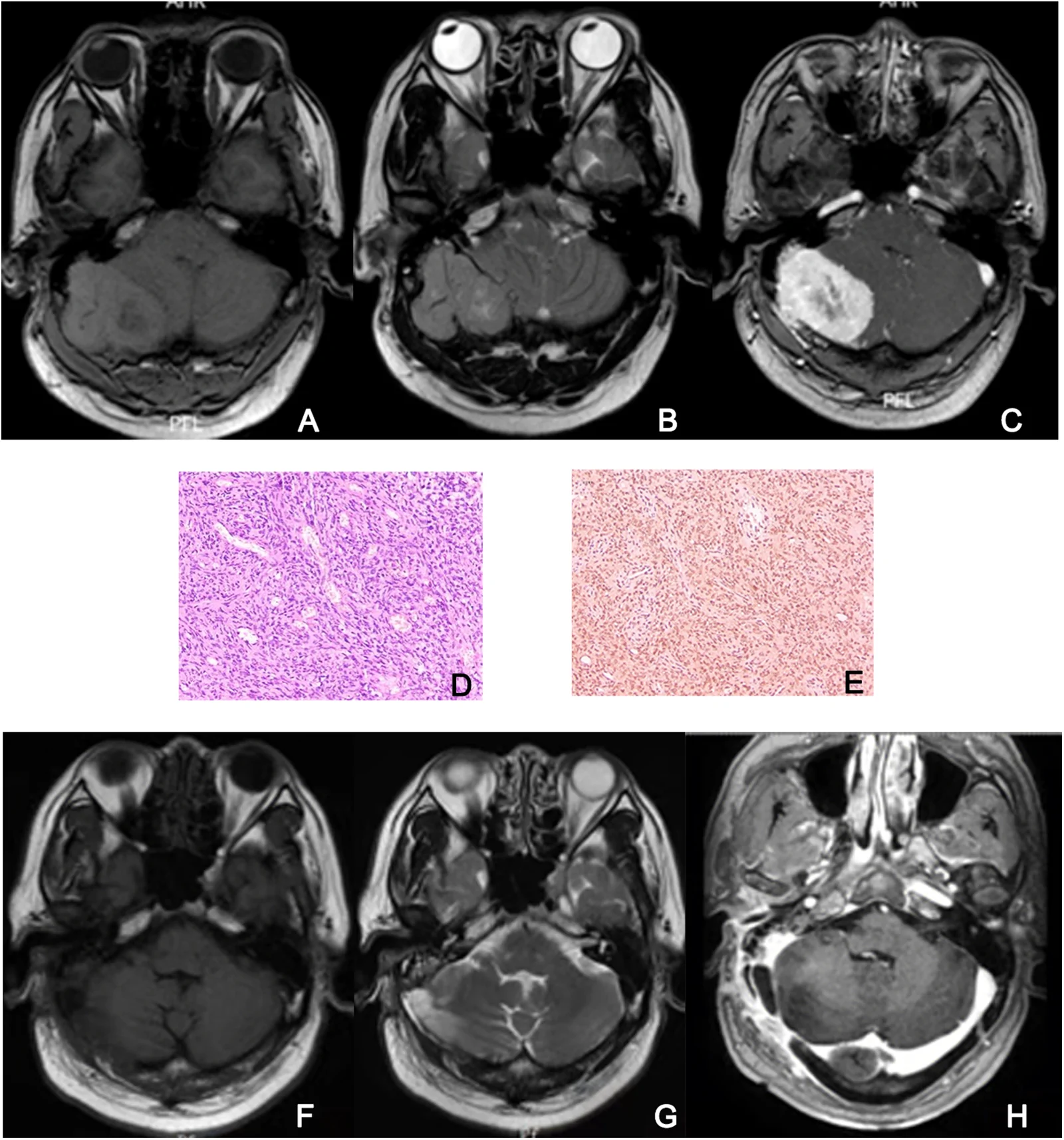
Preoperative and postoperative imaging findings and pathological characteristics of the patient. **(A–C)** Preoperative magnetic resonance imaging (MRI) revealed a round lesion in the right cerebellar hemisphere. The lesion demonstrated mild hypointensity on T1-weighted imaging (T1WI), heterogeneous signal intensity on T2-weighted imaging (T2WI), and heterogeneous enhancement following contrast administration. **(D,E)** Histopathological examination. Hematoxylin and eosin (H&E) staining and immunohistochemical analyses showed positivity for CD34, STAT6, Syn, and CD99, and negativity for NSE, EMA, GFAP, and PR. The Ki-67 labeling index was approximately 5%, and the MIB-1 positive control was positive(Scale:0.39). **(F–H)** Postoperative MRI follow-up demonstrated no evidence of tumor recurrence.

**Table 1:** Three-variable risk stratification model for predicting prognosis in solitary fibrous tumors.

	Patient 1		Patient 2	
Risk factor	Risk class	Score	Risk class	Score
Age	45	0	58	1
Tumor size (cm)	5.5	1	2.9	0
Mitotic count (/10 high-power fields)	>5	2	N/A	N/A
Total score	Low	3	Not assigned	Not assessable

Patient 2 was a 57-year-old female admitted on February 13, 2022, presenting with a 3-day history of sudden headache, dizziness, nausea, and vomiting. Physical examination showed intact consciousness, isocoric and round pupils with brisk light reflexes, normal muscle strength and tone in all four limbs, absence of pathological signs, and an absence of meningeal irritation signs. MRI revealed a well-circumscribed, round lesion in the left cerebellar hemisphere, showing a slightly hypointense signal on T1WI and a heterogeneous signal on T2WI. Post-contrast images revealed heterogeneous enhancement of the lesion, with internal non-enhancing hypointense areas and local attachment to the meninges. Additionally, mild enhancement of the surrounding nodules with ill-defined borders was observed (Figures 2A–C). The preliminary diagnosis was a space-occupying lesion in the left cerebellar hemisphere, with metastasis considered as a differential diagnosis. During surgery, the tumor base was found to be located at the transverse sinus. The lesion was dark red, firm in texture, richly vascularized, and closely adhered to the meninges. Histopathological examination revealed a highly cellular spindle cell tumor with marked hypercellularity, nuclear pleomorphism, elevated mitotic activity, and foci of hemorrhage and necrosis. Immunohistochemical findings supported the diagnosis of a SFT. The immunohistochemical profile demonstrated positivity for EMA, Vim, STAT6, Bcl-2, and CD99, with focal positivity for S-100, vascular positivity for CD34, and a Ki-67 proliferation index of approximately 30%. The tumor was negative for GFAP and PR. The final pathological diagnosis was a SFT (WHO Grade 3) (Figures 2D,E; Supplementary Document 2). The patient underwent adjuvant radiotherapy one month after surgical resection, and no recurrence was observed at the 1-year follow-up. (Figures 2F–H).

**Figure 2:**
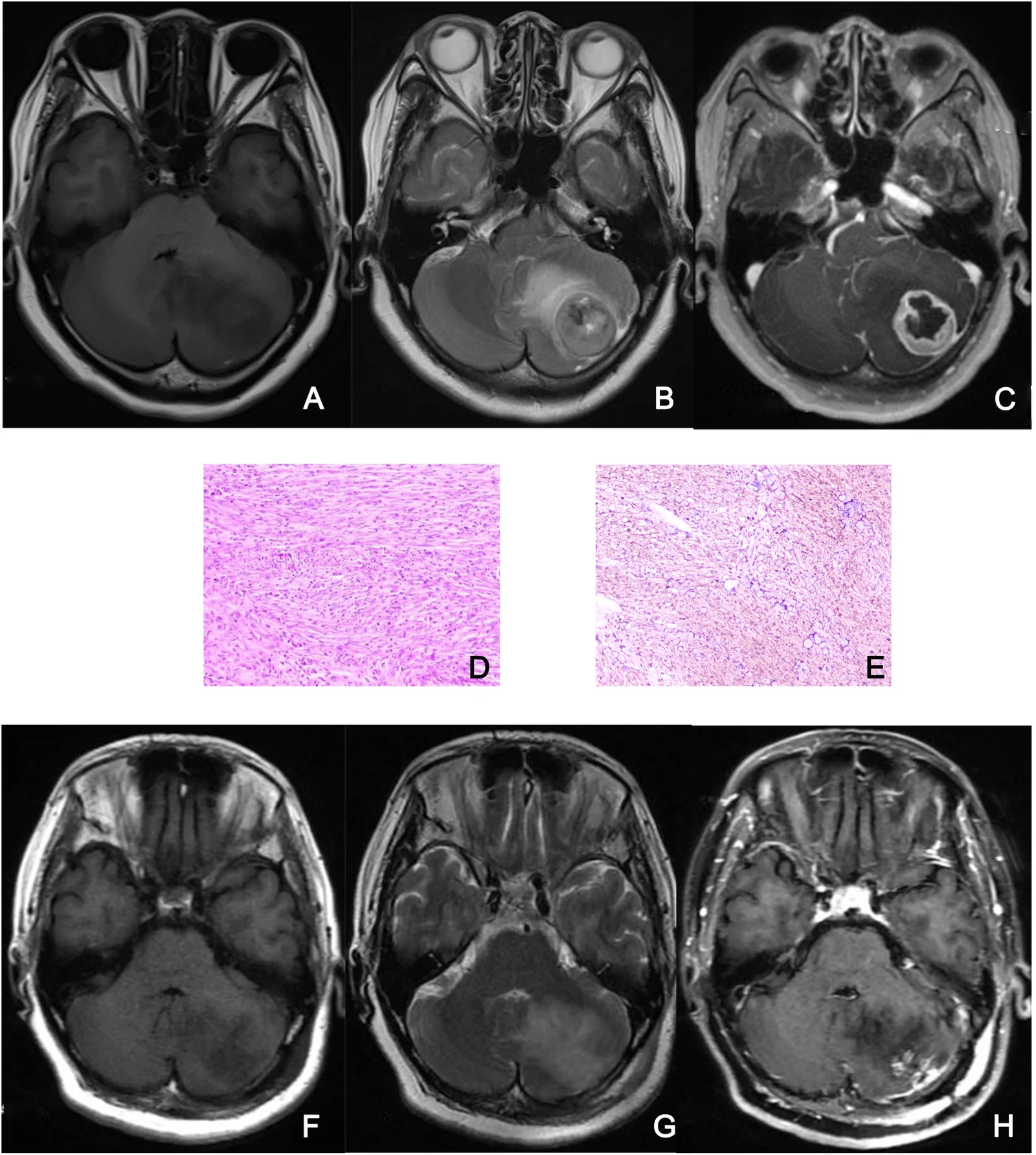
Preoperative and postoperative imaging findings and pathological characteristics of the patient. **(A–C)** Preoperative magnetic resonance imaging (MRI) revealed a round lesion in the left cerebellar hemisphere. The lesion exhibited slight hypointensity on T1-weighted imaging (T1WI), heterogeneous signal intensity on T2-weighted imaging (T2WI), and heterogeneous contrast enhancement. Non-enhancing hypointense areas were observed within the lesion, whereas mild enhancement was noted in the surrounding nodules with ill-defined margins. **(D,E)** Histopathological examination. Hematoxylin and eosin (H&E) staining and immunohistochemical analyses demonstrated positivity for S-100, EMA, STAT6, CD34, Bcl-2, and CD99, and negativity for GFAP and PR. The Ki-67 labeling index was approximately 30% (Scale:0.39). **(F–H)** Postoperative MRI follow-up demonstrated no evidence of tumor recurrence.

Both patients underwent gross-total resection (Simpson grade II). Given the CNS WHO grade 3 histology and the close relationship of both lesions to the dural venous sinuses, postoperative adjuvant radiotherapy was administered to the surgical bed with a safety margin, beginning approximately one month after surgery.

## Discussion

SFTs are rare spindle cell neoplasms of mesenchymal origin. Carneiro et al. first reported a meningeal SFT in 1996 (2), and the entity was subsequently integrated into the category of meningeal tumors in the 2007 World Health Organization (WHO) Classification of Tumors of the Central Nervous System. Historically, intracranial SFT and hemangiopericytoma (HPC) were both recognized as rare meningeal mesenchymal tumors sharing highly similar histological features and immunohistochemical staining profiles. Consequently, the 2016 WHO classification combined these entities under a single designation (SFT/HPC) and graded them as I, II, or III. In the updated 2021 fifth edition of the WHO classification, the term “hemangiopericytoma” was formally deprecated, and these tumors are now classified strictly as SFTs, assigned as CNS WHO grades 1, 2, or 3. High-grade (2 and 3) SFTs are characterized by rapid progression and a strong propensity for local recurrence and distant metastasis following surgical resection (3, 4). The clinical value of the present two cases lies in their demonstration of the diagnostic difficulties associated with cerebellar SFTs, the utility of integrating morphological and immunohistochemical parameters—particularly nuclear STAT6—and the importance of individualized surveillance and adjuvant treatment planning.

Early local recurrence or metastasis occurs in approximately 10% to 15% of high-grade cases (1, 5). Because the tumors of our two patients exhibited grade 3 features on histopathological evaluation and were thus associated with an elevated risk of recurrence, postoperative adjuvant radiotherapy was administered. Intracranial SFTs typically present in adulthood, showing no significant variance in distribution between male and female patients. The clinical manifestations lack pathognomonic specificity and are predominantly determined by tumor size, anatomic location, and histopathological grade. Mass effect and intracranial hypertension represent the primary clinical drivers of patient presentation, commonly manifesting as dizziness and headache. However, spontaneous intracranial hemorrhage has also been documented in the literature. Furthermore, approximately one-quarter of patients with systemic SFTs present with paraneoplastic syndromes, most notably refractory hypoglycemia—known as Doege-Potter syndrome—and digital clubbing (6).

Preoperative imaging evaluation, including head CT and MRI, is essential for determining tumor location, size, and relationship to surrounding neurovascular structures, thereby guiding surgical planning and preliminary diagnosis. However, misdiagnosis is common. In our study, Case 1 was diagnosed as a meningioma before surgery, whereas Case 2 was suspected to be a metastatic tumor; both were definitively diagnosed as SFTs via postoperative histopathological examination. Based on a synthesis of the imaging findings in these two cases and those reported in the literature, intracranial SFTs typically demonstrate isointense signals relative to the cerebral cortex on T1WI and heterogeneous, mixed high- and low-intensity signals on T2WI. Post-contrast images reveal heterogeneous enhancement. Because SFTs frequently originate from or closely adhere to the dura mater, contrast-enhanced MRI often exhibits the dural tail sign (7). The presence of mixed signals on T2WI, particularly in which the T2-hypointense areas show significant enhancement, is considered a characteristic MRI manifestation of intracranial SFTs (8, 9). The neuroimaging presentation of both patients in this report was consistent with these characteristic radiologic features.

Meningiomas, particularly fibrous meningiomas, are predominantly observed in middle-aged women and typically demonstrate uniform signal intensity on T2WI with homogenous contrast enhancement. Features such as intratumoral calcification, hyperostosis of the adjacent cranial bone, and a dural tail sign are more frequently associated with meningiomas than SFTs (7). Immunohistochemically, meningiomas are characterized by epithelial membrane antigen (EMA) expression and a lack of, or only focal, CD34 reactivity. The differential diagnosis of a dural-based cerebellar mass should include SFT, particularly when MRI demonstrates heterogeneous T2WI signal with marked enhancement of the hypointense components. In our two cases, the preoperative diagnoses were meningioma and metastasis, respectively, underscoring the need for heightened awareness of this entity.

Vestibular schwannomas typically arise within the cerebellopontine angle. Unlike dural-based SFTs, these neoplasms are centered on the internal auditory canal and frequently cause widening or erosion of the bony canal. MRI findings include low-to-mixed signal intensity on T1WI and predominantly high signal intensity on T2WI, reflecting frequent cystic degeneration.

Meningeal lymphomas and dural metastases represent the remaining critical differentials. On neuroimaging, meningeal lymphoma presents as a soft tissue mass involving the internal and external tables of the skull. It appears isodense on CT, hypointense to isointense relative to gray matter on T1WI, and hyperintense on T2WI, often accompanied by abnormal skull signals. Conversely, dural metastases typically occur in patients with a known history of primary malignancy. They characteristically present with diffuse, nodular, “cake-like” dural thickening and lack the distinct T2WI hypointense zones often seen in SFTs.

Regarding HPCs, these neoplasms have historically been considered a distinct entity from SFTs, albeit presenting with overlapping features, including a rich vascular supply, prominent staghorn-shaped capillaries, frequent T1WI flow voids, and significant peritumoral edema. Although the 2021 WHO classification has unified SFT and HPC under the single designation of SFT, distinguishing between these phenotypic variants is vital, given the high propensity of the latter for local recurrence and distant metastasis.

Notably, in cases where classic CD34 expression is limited, recent studies utilizing the stem cell marker ALDH1 or nuclear STAT6 expression aid in confirming SFT diagnosis, compensating for the limitations of the CD34 antibody (9). Nuclear STAT6 immunoreactivity, which results from the NAB2:STAT6 gene fusion, is currently regarded as the most reliable and specific diagnostic marker for SFT. This is particularly important in cases where CD34 expression is weak, patchy, or restricted to vascular endothelium, as observed in Case 2. Although EMA is typically negative or only focally positive in SFT, focal positivity may occasionally be encountered and should not be overinterpreted as evidence of meningioma. Similarly, S-100 is characteristically negative but may show focal reactivity in some cases. In Case 2, the combination of strong diffuse nuclear STAT6 staining, spindle-cell morphology, and a staghorn-like vasculature confirmed the diagnosis of SFT despite focal EMA and S-100 positivity and limited CD34 expression.

The diagnosis of intracranial SFT relies primarily on pathological examination, which provides the structural basis for the observed imaging characteristics. Macroscopically, these tumors typically exhibit irregular morphological configurations, including nodular, lobulated, or oval shapes (10). The tumor surface can be encapsulated, and the cut surface often appears gray or grayish-red when sliced open. Gross examination frequently reveals areas of focal hemorrhage or necrosis, with a tissue texture ranging from friable to firm. Under microscopy, the tumor displays abundant “staghorn-like” blood vessels and proliferating collagen fibers. Tumor cells tend to arrange in circular or concentric patterns around these vessels, demonstrating alternating hypercellular and hypocellular areas interspersed with collagen fibers. Integrating these detailed histological patterns with immunohistochemical findings (e.g., CD34 and STAT6 positivity) ensures definitive diagnostic accuracy (11, 12).

These histopathological features result in distinct MRI signal variations: dense collagen fibers exhibit low signal intensity on T2WI, whereas hypercellular areas produce high signal intensity (13). Immunohistochemical staining plays a pivotal role in confirming an SFT diagnosis; tumors typically show positivity for CD34, CD99, vimentin, and Bcl-2, and negativity for cytokeratin (CK), S-100, and EMA (14). A diagnosis of malignant intracranial SFT is considered when the Ki-67 labeling index exceeds 5%, or when the tumor exhibits hypercellularity, cellular pleomorphism, hemorrhage, necrosis, notable nuclear atypia, and brisk mitotic activity. Generally, malignant intracranial SFTs tend to display diminished or absent CD34 expression alongside elevated expression of p53, S-100, and Ki-67 (15). In the case of the female patient in this study, microscopic examination revealed increased cell proliferation and the presence of hemorrhage and necrosis. Additionally, her immunohistochemical profiles indicated high-grade malignancy, featuring a Ki-67 proliferation index of 30% along with focal S-100 positivity. These findings align with the described pathological criteria for high-grade tumors and correlate with their elevated propensity for recurrence and metastasis.

Most SFTs are well-demarcated from the adjacent brain parenchyma. Complete surgical resection currently represents the optimal therapeutic strategy, aiming to excise the mass and alleviate the neurological deficits induced by the mass effect. Thus, during tumor resection, it is essential to comprehensively evaluate the tumor's location, size, and boundary. Simultaneously, the extent of resection should be determined based on the tumor's vascularity and the patient's overall condition. This approach prioritizes patient safety over aggressive resection, which could potentially exacerbate the damage.

Regarding postoperative imaging surveillance, no formal consensus guidelines have been established specifically for intracranial SFTs. Nevertheless, based on the available literature, the following strategy is recommended. For CNS WHO grade 1 SFTs, a baseline MRI at 3–6 months postoperatively, followed by annual imaging for at least 5–10 years, and then every 2–3 years thereafter, given the documented potential for late recurrence even in low-grade lesions (1, 3). For CNS WHO grade 2 and 3 SFTs, a baseline MRI at 3 months postoperatively, followed by imaging every 6–12 months for at least 10 years, with continued long-term surveillance thereafter, given the substantial risk of local recurrence and extracranial metastasis. Recurrent disease or residual tumor following subtotal resection can be managed with stereotactic radiosurgery, such as Gamma Knife surgery. Evidence from several studies indicates that postoperative radiotherapy effectively reduces local recurrence rates and improves overall survival in patients with intracranial SFTs (4). Due to the highly vascular nature of SFTs, anti-angiogenic targeted therapies have been investigated internationally; however, the efficacy of these agents remains under investigation (16–18).

In conclusion, the incidence of intracranial SFTs is relatively low, and these neoplasms frequently present with nonspecific clinical manifestations, leading to potential misdiagnosis by clinicians unfamiliar with the condition. MRI characteristics typically include a solitary, solid mass with well-circumscribed margins. Distinctive neuroradiological features—specifically, the mixed high and low signal intensities on T2WI and the marked post-contrast enhancement of those underlying low-signal areas—are crucial to identifying the disease. However, definitive diagnosis relies on histopathological and immunohistochemical examination. The primary therapeutic approach consists of complete surgical resection followed by adjuvant radiotherapy. This treatment regimen yields a favorable prognosis for a subset of patients, though comprehensive management necessitates long-term surveillance.

## Data Availability

The raw data supporting the conclusions of this article will be made available by the authors, without undue reservation.
